# Dual time point imaging fluorine-18 flourodeoxyglucose positron emission tomography for evaluation of large loco-regional recurrences of breast cancer treated with electrochemotherapy

**DOI:** 10.2478/raon-2013-0054

**Published:** 2013-10-08

**Authors:** Louise Wichmann Matthiessen, Helle Hjorth Johannesen, Kristin Skougaard, Julie Gehl, Helle Westergren Hendel

**Affiliations:** 1 Center for Experimental Drug and Gene Electrotransfer (C^*^EDGE), Department of Oncology, Copenhagen University Hospital Herlev, Copenhagen University Hospital Herlev, Herlev, Denmark; 2 Department of Radiology, Copenhagen University Hospital Herlev, Herlev, Denmark; 3 Department of Clinical Physiology and Nuclear Medicine, Copenhagen University Hospital Herlev, Herlev, Denmark

**Keywords:** dual time point FDG PET, breast cancer, electrochemotherapy, response assessment, cutaneous metastases

## Abstract

**Background:**

Electrochemotherapy is a local anticancer treatment very efficient for treatment of small cutaneous metastases. The method is now being investigated for large cutaneous recurrences of breast cancer that are often confluent masses of malignant tumour with various degrees of inflammation. To this end 18-Flourine-Flourodeoxyglucose-Positron Emission Tomography/Computed Tomography (FDG-PET/CT) could be a method for response evaluation. However, a standard FDG-PET/CT scan cannot differentiate inflammatory tissue from malignant tissue. Dual point time imaging (DTPI) FDG-PET has the potential of doing so. The purpose of this study was to investigate if DTPI FDG-PET/CT could assess response to electrochemotherapy and to assess the optimal timing of imaging.

**Patients and methods:**

Within a phase II clinical trial 11 patients with cutaneous recurrences had FDG-PET/CT scans at three time points: 60 min, 120 min and 180 min after FDG injection. The scans were performed before and 3 weeks after electrochemotherapy.

**Results:**

A significant reduction in maximum standard uptake value at 60 min post injection was seen after treatment. Furthermore a change in the FDG uptake pattern was observed; from increasing uptake in up to 180 min post injection before treatment to stabilization of FDG uptake at 120 min post injection after treatment. The change in FDG uptake pattern over time lead to change of response in three target lesions; two lesions changed from stable metabolic disease to partial metabolic response and one lesion changed from partial metabolic response to stable metabolic disease. To ensure detection of the change in uptake pattern, scanning 60 and 180 min post injection seems optimal.

**Conclusions:**

The present study shows that FDG-PET/CT 60 and 180 min after tracer injection is a promising tool for response evaluation of cutaneous recurrences of breast cancer treated with electrochemotherapy.

## Introduction

**FIGURE 1.**
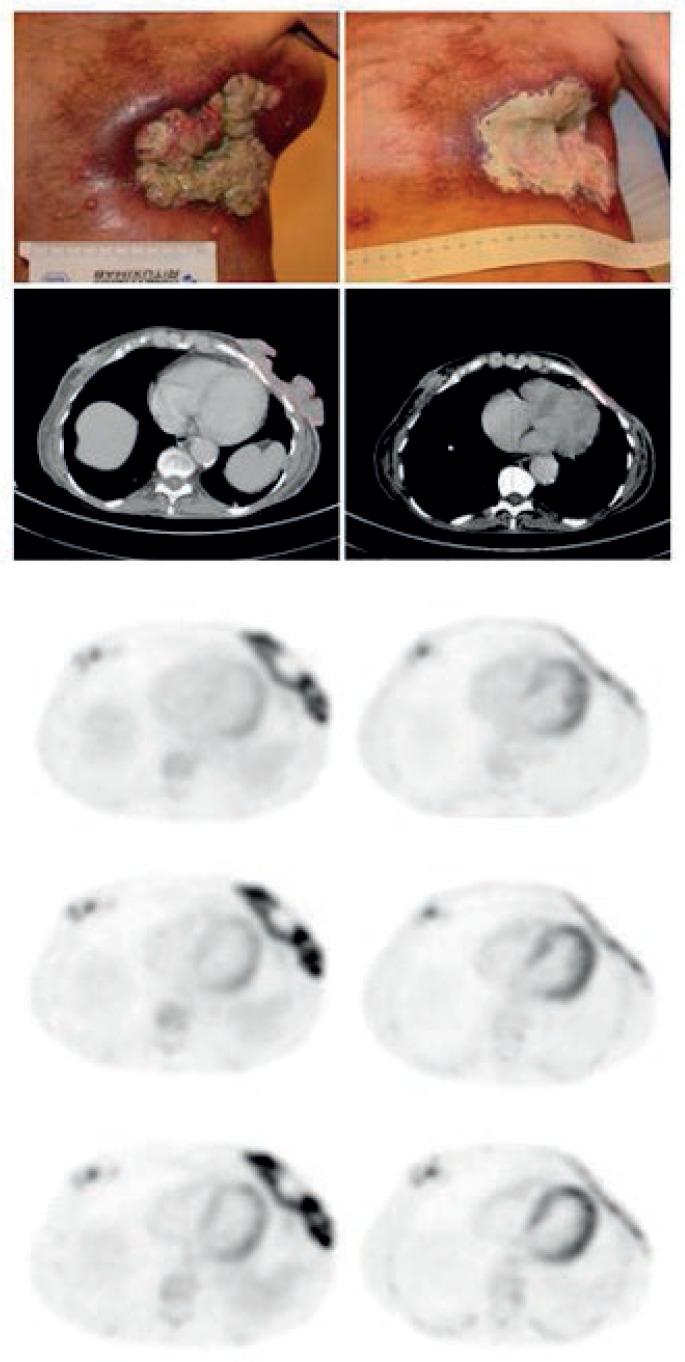
Large recurrence with varying depth and inflammation on the left chest wall of 68 y female. Left column shows from the top clinical presentation, CT-scan, PET scan at 60 min p.i., PET scan at 120 min p.i. and PET scan 180 min p.i. Right column. Same patients after one treatment. Change in SUVmax in target lesion at baseline compared to follow up was 29.7% at 60 min p.i., 71.2% at 120 min p.i., and 83.1% at 180 min p.i.

Could dual time point imaging (DTPI) fluorine-18 fluorodeoxyglucose positron emission tomography combined with computed tomography (FDG-PET/CT) be a useful tool for imaging of recurrent breast cancer treated with electrochemotherapy? The 5-year incidence of loco-regional recurrence of breast cancer is reported to be between 6% and 23% following mastectomy and approximately 6% after breast conserving surgery and radiotherapy.^1^ In spite of treatment, subsequent loco-regional recurrence occurs in 25–35% of patients 2 and of these it is reported that an estimated 30% will suffer from significant morbidity because of their local recurrence.^3^ Treatment of recurrent loco-regional breast cancer can be a clinical challenge. Local tumour control, in spite of any distant metastases, is important as the presence of uncontrolled loco-regional recurrence can cause severe patient distress due to large ulcerating and secreting tumours (Figure 1).

Electrochemotherapy is a local treatment using electric pulses to transiently permeabilize the cell membrane.^4^ It augments the effect of chemotherapy, by enabling passage over the cell membrane of otherwise non-permeating chemotherapeutic drugs.^5^ For the chemotherapeutic agent bleomycin, the effect is enhanced several hundred fold, enabling high efficacy after one or few treatments.^6^ Electrochemotherapy has proven highly effective in palliative treatment of cutaneous metastases less than 3 cm in diameter 7–13 and has shown promising results in terms of efficacy and alleviation of symptoms in heavily pre-treated breast cancer patients with loco-regional recurrence (Figure 1).^12^;^14^;^15^ Furthermore electrochemotherapy is currently being evaluated for deep seated tumours.^16^–^18^ Loco-regional recurrence of breast cancer that has previously been treated with radiotherapy and surgery is often a confluent mass of tumour and a varying degree of chronic and acute inflammation. Response evaluation of cutaneous loco-regional recurrences of breast cancer poses a challenge because inflammation cannot be distinguished from malignancy. Clinical evaluation with measurement of lesion extension has proven unsuitable for response evaluation after electrochemotherapy as it does not visualize deeper parts of the lesion and is not able to differentiate between inflammation, ulceration and tumour.^14^ Thus other methods for response evaluation of electrochemotherapy are needed.

FDG-PET/CT scan is a highly accurate method for staging of breast cancer recurrence.^19^;^20^ The technique provides metabolic information that can complement inconclusive findings derived from anatomical imaging and may better characterize disease extent. Furthermore, FDG-PET/CT can assess the response to ongoing therapy early as metabolic response prelude morphological changes. Uptake of FDG is however not specific to tumour cells as inflammatory cells also take up FDG.^21^ Studies have shown that the FDG uptake increases for several hours in malignant lesions, 21–23 which is rarely seen in inflammatory lesions and normal tissue.^24^ Thus, the difference in time course when using DTPI may improve the differentiation between inflammation, known to be present and viable malignant tumour.

In this study we investigated DTPI FDG-PET/CT as a possible method for response evaluation of loco-regional recurrent breast cancer treated with electrochemotherapy. This was done in order to evaluate if the method can assess treatment response to therapy and to determine the optimal timing of imaging FDG uptake in the loco-regional recurrence of breast cancer in this setting.

## Petients and methods

### Patients and treatment

In the period October 2008 to October 2010 patients with cutaneous loco-regional recurrence of breast cancer were prospectively accrued for a phase II investigator initiated electrochemotherapy protocol.^14^ Seventeen patients were included and treated within the protocol. Patients were separately asked if they would be willing to have DTPI FDG-PET/CT. Twelve patients completed DTPI FDG-PET/CT before (baseline) and after (follow up) electro-chemotherapy. DTPI FDG-PET/CT was performed median 5 days (range, 2–15) before electrochemotherapy and 24 days (range, 17–59 days) after electrochemotherapy. Eleven patients were evaluable while one patient was not evaluable due to lack of contrast enhancement and lack of metabolic activity in the target lesions, making them indistinguishable from the surrounding tissue.

Electrochemotherapy was considered when no other treatment options were available and patients had symptoms from large (> 3 cm) cutaneous loco-regional recurrence of breast cancer. All patients gave informed written consent and the protocol was approved by the Regional Research Ethics Committee, The Danish Medicines Agency (clinicaltrials.gov identifier: NCT00744653), and the Data Protection Agency.

Electrochemotherapy was performed according to the standard operating procedures.^25^ General anaesthesia was induced in all patients. Bleomycin was administered intravenously using a standard dose of 15.000 IU/m^2^.

### Data acquisition

#### Clinical examination

The cutaneous loco-regional recurrences to be treated were defined as target lesions. According to protocol loco-regional recurrences smaller than 3 cm were not considered target lesions.

Clinical examination was performed at baseline and at follow up. The longest diameter of the target lesions was recorded using a ruler and verified with digital photography.

### FDG-PET/CT-scanning

A combined FDG-PET 60 min post-tracer injection (p.i.) and contrast enhanced CT scan of the thorax was performed in each patient at baseline and at follow up. Furthermore, PET scanning was performed 120 min p.i. and 180 min p.i at baseline and at follow up. Patients fasted for at least 6 hours before the examination. A blood sample determined glucose level (Ascension Contour, Bayer Health Care, Germany) before FDG administration to ensure the inclusion criteria of euglycemia (concentrations below 120 mg/dL). All examinations were performed with a Gemini PET/CT system (Philips Medical, The Netherlands), consisting of a dedicated germanium oxyorthosilicate full-ring PET scanner and a dual-slice helical CT scanner. After intravenous injection of 18F-FDG (370 MBq), the patients rested in a quiet room for 45 min. The scans were performed with the patients in supine position with arms over the head and were initiated with a low-dose CT scan (20 mA, 140 kV, 512 × 512 matrix) covering the thorax and used for attenuation correction. Thereafter, emission measurements were performed in the 3-dimensional mode with a 144 × 144 matrix (60 min p.i.). Emission scan time per bed position was 2 min; 4 bed positions (field of view: 155 mm) were acquired. After the first emission scan, a diagnostic CT scan of the thorax was performed after automated intravenous injection (100 mL 5 mL/sec) of iodine-containing contrast medium (Omnipaque 350 mg/ml, GE Healthcare Deutschland GmbH). Scan delay after injection was 50 seconds. Diagnostic dual-slice CT with an axial field of view of 600 mm and a matrix of 512 × 512 was performed in the spiral mode under continuous acquisition at 120 kV and 145 mA (slice thickness of 5.0 mm, increment of 2.5 mm/seconds, rotation time of 0.5 seconds, and pitch index of 1). The patients were then resting in comfortable chairs in the waiting room for two further emission scans of the thorax at 120 and 180 min p.i. The average examination time (including rest periods) was approximately 4 hours. Total time spent in the scanner was approximately 30 min.

### Response evaluation

#### Clinical Evaluation:

Assessment of tumour response in target lesions was done in accordance with RECIST 1.0: 26 Complete response (CR) was defined as disappearance of the target lesion, partial response (PR) was defined as at least 30% decrease in the diameter of the target lesion, progressive disease (PD) was defined as at least 20% increase in the diameter of the target lesion and stable disease (SD) as neither sufficient shrinkage to qualify for PR or sufficient increase to qualify for PD. Non-target lesions and systemic disease was not addressed.

### FDG-PET/CT-scans

CT scans were analysed by an experienced radiologist using Eclipse Cone Planning version 8.9 (Varian Medical Systems Inc., California, USA), standardly used for radiotherapy planning. The largest diameter of target lesions in the axial plane was recorded and the sum of the diameters evaluated in accordance to RECIST.^26^

PET data were reconstructed iteratively with a method based on the row action maximum-likelihood algorithm (RAMLA) using the PETView software (Philips Medical, The Netherlands). Image analysis and measurements were performed by a specialist in nuclear medicine in collaboration with the oncologist responsible for the treatment. Tumour tracking in the Brilliance Workspace soft ware package (Philips, The Netherlands) was used. This allowed simultaneous viewing and analysis of the PET scans acquired at 60, 120 and 180 min p.i. Scans at baseline and follow up were analysed separately. The FDG uptake in target lesions were assessed semiquantitatively by measuring the maximum standardized uptake value (SUVmax) within a three dimensional ellipsoidal volume of interest (VOI) covering the target. SUV’s normalized to body weight were automatically drawn by isocon-touring in the selected VOI. SUV of 2.5 was used as cut off for the isocountoring. Background activity was determined as the SUVmax in the aortic arch using a spherical VOI with a diameter of 1.2 cm. Lesions outside the treated area and new lesions were not considered in this study. SUVmax in each target lesion was recorded at baseline and at follow up at 60, 120 and 180 min p.i. A complete metabolic response (CMR) was considered as visual disappearance of tumour activity in the target lesion, so that it became indistinguishable from surrounding normal tissue. Partial metabolic response (PMR) was considered when more than a 25% decline in SUVmax was observed, progressive metabolic disease (PMD) was considered when more than a 25% increase in SUVmax was observed and stable metabolic disease (SMD) was defined as not CMR, PMR or PMD as suggested limits in the EORTC criteria.^27^

### Statistics

All of the quantitative values were expressed in terms of mean ± SD. A paired t-test was applied to determine the difference in SUVmax at baseline and at follow up. Repeated measurement analysis using general linear model was applied to determine the difference in SUVmax at 60, 120, and 180 min p.i. A p value of <0.05 was considered significant. Statistical analyses were performed using SPSS for windows version 16.0.

## Results

### Patients

The eleven evaluable patients with cutaneous loco-regional recurrence of breast cancer presented with a total of 16 target lesions (median 1 lesion per patient, range, 1–5). Baseline DTPI FDG-PET/CT was performed five days (range, 2–15) before electro-chemotherapy and follow up DTPI FDG-PET/CT was performed 23 days (range, 17–35 days) after electrochemotherapy.

### Clinical evaluation

Before treatment the mean tumour diameter was 8.5 ± 6.3 and after treatment the mean tumour diameter was 7.0 ± 5.8 cm with a mean difference of 1.5 ± 2.5 cm (p=0.024). Observed responses were CR in two lesions, PR in three lesions, SD in 10 lesions, and PD in one lesion, resulting in objective response in 5 out of 16 lesions (31%) (Table 1).

### CT-scans

Axial diameter and volume of the treated lesions were measured at baseline (median 5 days (range, 2–15) before electrochemotherapy) and at follow up (24 days (range, 17–59 days) after electrochemotherapy). Mean tumour diameter at baseline was 8.2 ± 6.0 cm and at follow up 6.4 ± 5.5 cm with a mean difference of 1.8 ± 2.9 cm (p=0.026).

Observed responses were CR in 2 lesions, PR in 7 lesions, SD in 5 lesions, and PD in 1 lesion, resulting in objective response of 9 out of 16 lesions (56%) (Table 1).

### PET-scans

#### Baseline PET

At baseline (median 5 days (range, 2–15) before electrochemotherapy) mean SUVmax in target lesions was 8.7 ± 6.1 at 60 min p.i., 11.0 ± 8.2 at 120 min p.i., and 13.4 ± 9.2 at 180 min p.i. The corresponding mean SUVmax for background was 1.7 ± 0.3 at 60 min p.i., 1.4 ± 0.2 at 120 min p.i., and 1.3 ± 0.4 at 180 min p.i. (Table 2). Mean percentage change in SUVmax in target lesion between 60 and 120 min p.i. was 22.5 ± 14.7% (p=0.002), between 60 and 180 min p.i. 41.1 ± 29.6% (p=0.001), and between 120 and 180 min p.i. 15.3 ± 24.4% (p=0.001). The corresponding percentage change in SUVmax for background was −16.3 ± 12.5% (p=0.004), −27.3 ± 29.7% (p=0.058), and −9.0 ± 40.7% (p=1.000) (Table 2).

#### Follow-up PET

At follow up (24 days (range, 17–59 days) after electrochemotherapy) mean SUVmax in target lesion was 5.7 ± 5.0, 7.2 ± 6.9, and 7.3 ± 6.8 at 60, 120, and 180 min p.i. respectively. The corresponding mean SUVmax for background was 1.8 ± 0.4, 1.5 ± 0.4, and 1.4 ± 0.5 (Table 2). Mean percentage change of SUVmax in target lesions between 60 and 120 min p.i. was 30.3 ± 16.8% (p=0.008) between 60 and 180 min p.i. 36.8 ± 41.1% (p=0.052), and between 120 and 180 min p.i. −1.3 ± 32.5% (p=1.000). The corresponding change in SUVmax for background was −16.3 ± 16.7% (p=0.019), −24.2 ± 24.6% (p=0.059), and −7.0 ± 33.7% (p=1.000) (Table 2).

#### Change between baseline and follow-up PET

The mean changes in SUVmax between baseline and follow-up scans were 3.1 ± 3.1, 3.8 ± 3.8, and 5.9 ± 6.0 at 60, 120, and 180 min p.i. respectively. Mean percentage changes of SUVmax between baseline and follow up scans were 38 ± 38% (p=0.001), 37 ± 47% (p=0.001), and 47 ± 46% (p=0.002) at 60, 120 and 180 scans respectively.

**FIGURE 2.**
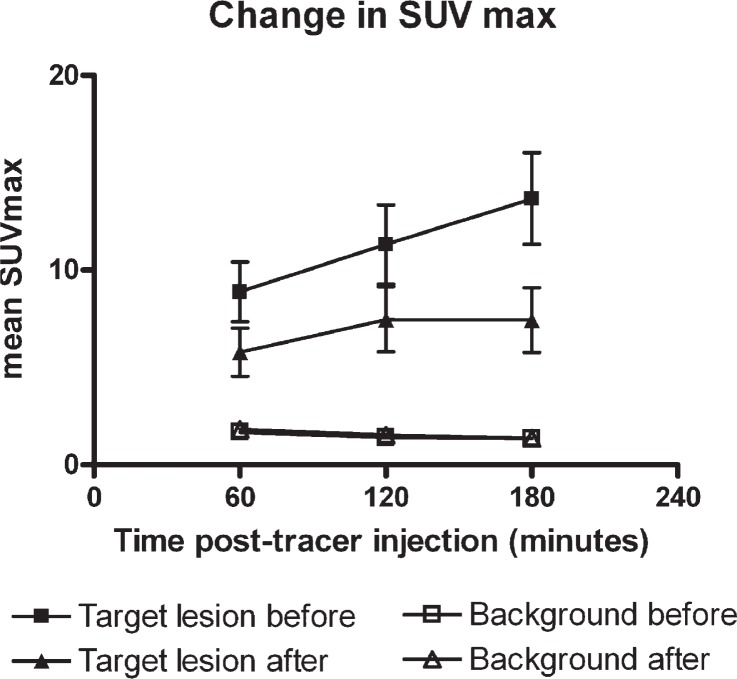
Change in SUVmax over time in target lesions and background. There was significant increase in mean SUVmax over time in target lesions before treatment wheras the mean SUVmax in target lesions after treatment stabilized at 120 min after FDG injection. Mean SUVmax in background (measused in the aortic arch) did not increase over time.

At the 60 min p.i. scans CMR was observed in 2 lesions, PMR in 7 lesions, SMD in 6 lesions, and PMD in 1 lesion. The corresponding responses observed at the 120 min p.i. scans were CMR in 2 lesions, PMR in 7 lesions, SMD in 6 lesions, and PMD in 1 lesion. Observed responses at the 180 min p.i. was CMR in 2 lesions, PMR in 9 lesions, SMD in 3 lesions, and PMD in 1 lesion (Table 1). One patient did not have the baseline 180 min post tracer injection scan and was therefore not evaluated at 180 min p.i. The changes in SUVmax over time are illustrated in Figure 2 and an example is given in Figure 1.

## Discussion

In this study of 11 patients with cutaneous loco-regional recurrent breast cancer, 16 lesions where treated with electrochemotherapy and evaluated with DTPI FDG-PET/CT. We observed two changes between the baseline and follow-up PET-scan: a significant reduction in SUVmax 60 min p.i. after electrochemotherapy (standard imaging protocol) and a change in the SUVmax pattern from a steadily increasing SUVmax in up to 180 min before electrochemotherapy to a stabilization at 120 min p.i. on the after electrochemotherapy, making the difference in SUVmax before and after electrochemotherapy more pronounced using the 180 min p.i. scan. A significant reduction of SUVmax 60 min p.i is in routine anti-cancer treatment monitoring used as a surrogate parameter for tumour response and we interpret it as response to the electrochemotherapy in our study. This interpretation is strongly supported by the change in uptake pattern: after treatment the SUVmax stabilizes after 120 min in contrast to the baseline scans, where there is a steadily increased uptake in up to 180 min. This might indicate more tissue with inflammatory cells than with malignant cells at follow up as the SUVmax reaches a plateau in inflammatory cells faster than in malignant cells.^22^ This perception is in accordance with Boerner *et al.*^28^ who recommend a three hour protocol for detection of breast cancer and Caprio *et al.*^29^ who observed an increase SUVmax over time in primary breast malignancies using DTPI FDG-PET/CT. We observed continuous uptake of FDG in target lesions at baseline scans for at least three hours suggesting 180 min p.i. could be the optimal time for imaging of FDG uptake in lesions such as cutaneous loco-regional recurrence of breast cancer. The difference between SUVmax at baseline and follow-up was significantly larger at the 180 min p.i. scans compared to the 60 min p.i. scans. Therefore the 180 min p.i. scans might enable visualization of smaller differences in SUVmax changes compared to the 60 min p.i. scans and provide useful information in the event that re-treatment needs to be planned.

Clinical examination with measurement of lesion extension is used for evaluation of electrochemotherapy and was also done in this study. CR was observed in two target lesions, PR in three, SD in 10 lesions and PD in one lesion using this method. It is difficult to measure the extent of the lesions in a clinical setting. The FDG-PET/CT scans showed a different picture from the clinical evaluation with more patients having partial response than patients having stable disease.^14^ This illustrates that clinical evaluation of response in highly heterogeneous lesions such as a loco-regional recurrence of breast cancer treated with electrochemotherapy may not be suitable and that FDG-PET/CT may be more precise methods for response evaluation. CT-scans can illustrate the anatomical extent of the lesions but may include scar tissue, inflammatory tissue and thickened skin that cannot be differentiated from malignant tissue. PET-scans help in this differentiation by adding biological important information of metabolic activity to CT-scans.

Other forms of PET scanners such as the Positron Emission Mammography has shown high accuracy and appears useful in biopsy guidance, surgical planning and surveillance for recurrence 30, but is unsuitable for patients with cutaneous loco-regional recurrence of breast cancer as most of these have had mastectomy.

With DTPI PET the time course of FDG uptake can be used to differentiate inflammation from malignancy which further improves the evaluation of response. The change in FDG uptake over time did change the response in three target lesions; in two lesions from SMD to PMR and in one lesion from PMR to SMR compared to the 60 min scan. To confirm our observations further studies could include biopsies, to determine the extent of inflammation and malignant tissue after treatment. Induction of inflammatory papules in the skin could also be of interest in evaluating the control time course of FDG uptake in inflammatory tissue. This was not done in this primary study.

Although DTPI FDG-PET/CT is an experimental, expensive and time consuming investigation not favourable for routine clinical application, we do find it a promising non-invasive method for response evaluation of cutaneous loco-regional recurrent breast cancer treated with electrochemotherapy. Furthermore it can be of use in deep seated tumours 31;32, in the neoadjuvant setting^33^ and investigated for other local treatments such as irreversible electroporation^34^ or radio-frequency ablation as well. Carbon-11-thymidine has shown to be promising in measurement and prediction of early response to chemotherapeutic agents and could also be considered in these types of treatments as it is not taken up by inflammatory cells.

In conclusion, this study indicates that not only FDG-PET/CT but also DTPI FDG-PET/CT is promising for evaluation and planning of electrochemotherapy and could be useful for other localized anti-cancer treatments as well. Although further studies are needed, a planned scan time at 180 min would be feasible and could add important information in particular when inflammation and cancer are superposed.

## Figures and Tables

**TABLE 1.** Response evaluation with clinical examination, CT and PET/CT-scans

**Patient no (lesion no)**	**Clinical response evaluation**			**Response evaluation on CT scans**			**Response evaluation with PET scans 60 min post tracer injection.**			**Response evaluation with PET scans 120 min post tracer injection.**			**Response evaluation with PET scans 180 min post tracer injection.**		

	**Base-line (cm)**	**Follow up (cm)**	**Response**	**Base-line (cm)**	**Follow up (cm)**	**Response**	**Baseline SUV-max 60 min**	**FUP SUV-max 60 min**	**Response**	**Baseline SUV-max 120 min**	**FUP SUV-max 120 min**	**Response**	**Baseline SUV-max 180 min**	**FUP SUV-max 180 min**	**Response**
1^*^	7.5	6.3	SD	10.6	6.3	PR	2.6	2.4	SMD	3.1	2.4	SMD	ND		
2 (a)	3.7	0.0	CR	1.4	0.0	CR	0.6	0.0	CMR	0.8	0.0	CMR	0.6	0.0	CMR
2 (b)	3.2	0.0	CR	0.7	0.0	CR	0.7	0.0	CMR	0.6	0.0	CMR	0.5	0.0	CMR
3	6.5	6.5	SD	1.0	1.3	PD	1.6	2.1	PMD	1.5	3.0	PMD	2.6	4.5	PMD
4	10.0	10.0	SD	11.0	10.5	SD	22.6	19.3	SMD	30.7	25.6	SMD	34.9	23.4	PMR
5 (a)	12.0	9.5	SD	11.8	9.1	SD	11.7	9.6	SMD	16.6	14.8	SMD	19.9	14.0	PMR
5 (b)	3.5	2.5	SD	3.9	2.7	PR	11.4	4.0	PMR	14.1	5.5	PMR	17.5	6.6	PMR
5 (c)	3.5	2.5	SD	3.9	1.2	PR	8.3	2.3	PMR	10.3	3.2	PMR	12.7	3.4	PMR
5 (d)	6.0	5.0	SD	7.1	4.3	PR	9.6	9.2	SMD	13.6	11.9	SMD	15.6	12.5	SMD
5 (e)	3.5	1.5	PR	4.2	1.2	PR	10	2.3	PMR	12.8	3.2	PMR	14.2	3.0	PMR
6	9.0	11.0	PD	10.0	14.8	PD	11.8	10.9	SMD	15.9	14.9	SMD	16.6	16.5	SMD
8	11.0	12.0	SD	11.9	8.0	PR	6.7	4.7	PMR	7.9	2.3	PMR	9.1	1.5	PMR
9	3.2	3.2	SD	2.4	2.5	SD	5.0	2.8	PMR	6.5	3.2	PMR	6.7	3.2	PMR
10	25.0	16.5	PR	19.7	10.8	PR	5.6	4.6	SMD	7.1	5.6	SMD	10.5	5.6	PMR
12	9.0	5.5	PR	15.2	16.0	SD	17.9	8.9	PMR	21.8	10.3	PMR	25.0	3.9	PMR
15	20.0	20.0	SD	16.3	13.7	SD	12.4	8.1	PMR	13.5	9.2	PMR	13.9	10.8	SMD

**Mean**	8.5	7.0		8.2	6.4		8.7	5.7		11.0	7.2		13.4	7.3	
**S.D.**	6.3	5.8		6.0	5.5		6.1	5.0		8.2	6.9		9.2	6.8	

Responses recorded for single lesions evaluated with clinical evaluation, CT and PET/CT scans at 60, 120 and 180 minutes after FDG injection are presented. For patient number 4 and 10 the observed response evaluated with PET/CT changes from SMD at 60 and 120 min to PMR at 180 min. For patient number 8 the observed response evaluated with PET/CT changes from SMD at 60 min to PMR at 120 and 180 min. (CMR: complete metabolic response, PMR: Partial metabolic response, SMD: Stable metabolic disease, PMD: progressive metabolic disease). ND: Not done

Patient numbers refers to numbers given consecutively when patients were included in the trial.

* Scan at 180 min was not performed at baseline

**TABLE 2.** SUVmax values before and after treatment

	60 min	120 min	180 min	RI_120–60_	RI_180–60_	RI_180–120_
Before treatment (n = 16)	8.7 ± 6.1	11.0 ± 8.2	13.4 ± 9.2	22.5% ± 14.7 %	41.1% ± 29.6 %	15.3% ± 24.4%
Background before treatment (n=11)	1.7 ± 0.3	1.4 ± 0.2	1.3 ± 0.4	−16.3% ± 12.5%	−27.3% ± 29.7%	−9.0% ± 40.7%
After treatment (n =16)	5.7 ± 5.0	7.2 ± 6.9	7.3 ± 6.5	30.3% ± 16.8%	26.8% ± 41.1%	−1.3% ± 32.5%
Background after treatment (n =11)	1.8 ± 0.4	1.5 ± 0.4	1.4 ± 0.5	−16.3%± 16.7%	−24.2% ± 24.6%	−7.0% ± 33.7%

Table 2 shows the SUVmax values for lesions before and after treatment with electrochemotherapy. The corresponding values for background measured as SUVmax in a 1.2 cm spherical ROI in the Aortic arch is also shown. Eleven patients with 16 target lesions were scanned. All data are presented as mean ± S.D.

RI: Retention index.; RI_120–60_: (SUV_60min_-SUV_120min_)/SUV_60min_×100%; RI_180–60_: (SUV_60min_-SUV_180min_)/SUV_60min_×100%; RI_180–120_: (SUV_120min_-SUV_180min_)/SUV_120min_×100%
